# Development and Applications of a Comprehensive Land Use Classification and Map for the US

**DOI:** 10.1371/journal.pone.0094628

**Published:** 2014-04-11

**Authors:** David M. Theobald

**Affiliations:** 1 Conservation Science Partners, Inc., Fort Collins, Colorado, United States of America; 2 Department of Fish, Wildlife, and Conservation Biology, Colorado State University, Fort Collins, Colorado, United States of America; University of California, Berkeley, United States of America

## Abstract

Land cover maps reasonably depict areas that are strongly converted by human activities, but typically are unable to resolve low-density but widespread development patterns. Data products specifically designed to resolve land uses complement land cover datasets and likely improve our ability to understand the extent and complexity of human modification. Methods for developing a comprehensive land use classification system are described, and a map of land use for the conterminous United States is presented to reveal what we are doing on the land. The comprehensive, detailed and high-resolution dataset was developed through spatial analysis of nearly two-dozen publicly-available, national spatial datasets – predominately based on census housing, employment, and infrastructure, as well as land cover from satellite imagery. This effort resulted in 79 land use classes that fit within five main land use groups: built-up, production, recreation, conservation, and water. Key findings from this study are that built-up areas occupy 13.6% of mainland US, but that the majority of this occurs as low-density exurban/rural residential (9.1% of the US), while more intensive built-up land uses occupy 4.5%. For every acre of urban and suburban residential land, there are 0.13 commercial, 0.07 industrial, 0.48 institutional, and 0.29 acres of interstates/highways. This database can be used to address a variety of natural resource applications, and I provide three examples here: an entropy index of the diversity of land uses for smart-growth planning, a power-law scaling of metropolitan area population to developed footprint, and identifying potential conflict areas by delineating the urban interface.

## Introduction

The majority of the global biosphere is occupied by human-modified landscapes of agricultural and urban land uses, with less than 20% semi-natural and about 25% “wild” [1]. Because of the extent of human domination, it is globally important to understand the extent and effects of human use of ecosystems [2]. Various aspects have been linked directly to land uses, ranging from the consequences on biodiversity [3], water security [4], and human health [5]. Yet for regional to national extents land use is typically measured and mapped at a coarse spatial resolution (e.g., state or county unit) and for only broad categories of use (e.g., urban vs. agriculture). Some land cover maps depict “developed” or built-up land cover types, but these typically miss low-density development that is either diffuse but has direct and permanent human activities or obscured by canopy cover [6]. This under-representation has been estimated to be roughly twice the extent of built-up land cover in the US [7]. Consequently, data products specifically designed to resolve different land use types and patterns would complement land cover datasets and likely improve our ability to understand the extent and complexity of human modification.

In the US a few efforts have developed land use/cover datasets that typically provide more detail than global approaches [8]. These include the National Land Cover Dataset (NLCD [9]; the USGS Land Cover Trends project [10]; the US Department of Economic Research Service (ERS) that summarizes major land uses [11]; and the Natural Resource Conservation Service's National Resource Inventory (NRI) [12]. Generally, these efforts have focused on characterizing land use/cover in rural areas and have not sought to distinguish land use types in more urban and developed areas (but see [13]). The NRI and ERS provide helpful land use data but at county to state levels and for only very coarse classes (i.e. urban) on private lands. The NLCD provides high spatial resolution (∼0.2 ha) but predominately is based on cover, not use [9], [10], and is based on the Anderson classification system [14]. Two notable recent efforts have produced an approach to develop a prototype for a national land use product targeted for urban land uses and derived principally from NLCD for US urban areas [15] and combined land use/cover approach for Canadian cities [16]. In short, we lack a detailed, consistent, spatially-explicit dataset that directly maps the patterns of *land use* in the United States.

In this paper I describe the generation and applications of this new, detailed, and high-resolution land use dataset. In doing so, two dichotomies are important to consider. The first is the distinction between land *use* and land *cover*. Indeed, a major challenge for “land change” science is to develop systems for monitoring and observing land use and land use change [17], and a critical aspect to ensure progress is to clearly distinguish land use from land cover [18], [19]. Although the terms land use and land cover are often used interchangeably, land use is quite distinct from cover. Land use and land cover typically are defined using a systems description where human driving forces lead to actions or activities that are characterized by a land use [19]. A land use may in turn manipulate land cover to such an extent that it results in conversion (e.g., cropland to residential) or modification (e.g., fertilization of cropland). *Land cover* is defined as the observed bio-physical cover on the earth's surface, whereas *land use* is characterized by the arrangements, activities and inputs of people to produce, change or maintain a land cover. For example, grassland is a cover type, but rangeland is a use.

The second dichotomy is the difference between spatial data on land use vs. the system used to classify the land uses. Surprisingly, few efforts have focused specifically on developing land *use* classification systems, but there are two notable exceptions. US urban planners developed the Land Based Classification Standards [20] to map detailed, parcel-level land uses appropriate mostly for urban settings, and it distinguishes activity, function, structure, site, and ownership characteristics. The Australian Land Use Map [21] has been developed over the past 15 years as a hierarchical structured classification to map the primary land use, typically from interpretation of aerial photography. At the highest level there are six classes: conservation, production from natural environments, dryland agriculture, irrigated agriculture, built-up uses, and water.

My goal in this paper is to describe a comprehensive, detailed, consistent, and high-resolution dataset that characterizes human activities on the landscape — that incorporates and maps all sectors of use: agricultural, stewardship/conservation, and urban. To do this, I: a) created a hierarchical land use classification system; b) constructed a detailed land use map built from nationally-available datasets through application of a set of logical rules; c) compared the general patterns to standard statistical summaries (i.e. NRI) and verified with a sample-based, detailed land use dataset; d) and summarized the national and regional patterns and configurations of various land uses. The general intent of this work is to provide both a consistent and comprehensive land use classification system as well as a new, spatial dataset that provides fundamental information on the primary human activities occurring in the US. This dataset provides a significant improvement in our ability to understand land use patterns, which will have broad utility for natural resource applications.

## Materials and Methods

### Classification system

Ideally, a classification system should be applicable over broad extents, contain hierarchical classes that would allow aggregation and a comprehensive range of classes so that all areas fit within a class; result in repeatable and consistent results through time and by interpreter; and have reasonably-balanced levels of accuracy amongst classes [14]. Adhering to these criteria and building on other classification systems, I synthesized a variety of classification systems [9], [12], [13], [14], [20] to construct a three-level hierarchical system that is comprehensive, consistent, and exclusive. The first level is based on the major use groups of built-up, production, recreation, and conservation. This broadly captures the four main ways that humans interact with their environment: by constructing buildings and living and working at a location (built-up), producing or removing natural resources from an area such as cropland agriculture, grazing, or mining (production), visiting an area for the purposes of enjoyment, but not building significant structures or removing resources (recreation and tourism), or by setting aside places primarily to protect natural systems and wildlife habitat (conservation).

**Figure 1:**
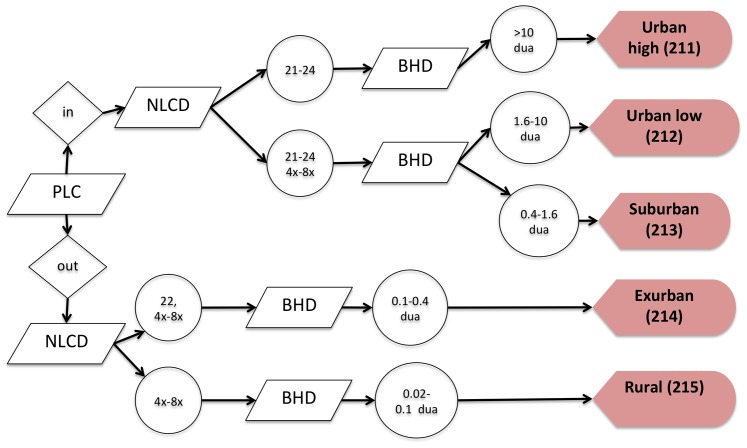
A flow chart depicting the process to map the residential land uses. Locations mapped as high-density urban residential were mapped in the National Land Cover Dataset 2006 (NLCD) as a built-up cover type and had a census block housing density in 2010 (BHD) of at least 10 dwelling units per acre (dua). Low-density urban residential was mapped as within PLC, on land but not water cover types (NLCD water, barren, or wetlands), and had a housing density between 1.6 and 10 dua. Suburban residential was mapped similar to low-density urban but at a density between 0.4 and 1.6 dua. Low-density residential areas were required to be outside of cities/towns (and were defined using Census places; PLC), and exurban residential had a housing density between 0.1 and 0.4 dua, while rural residential had a housing density between 0.025 and 0.1 dua.

**Figure 2:**
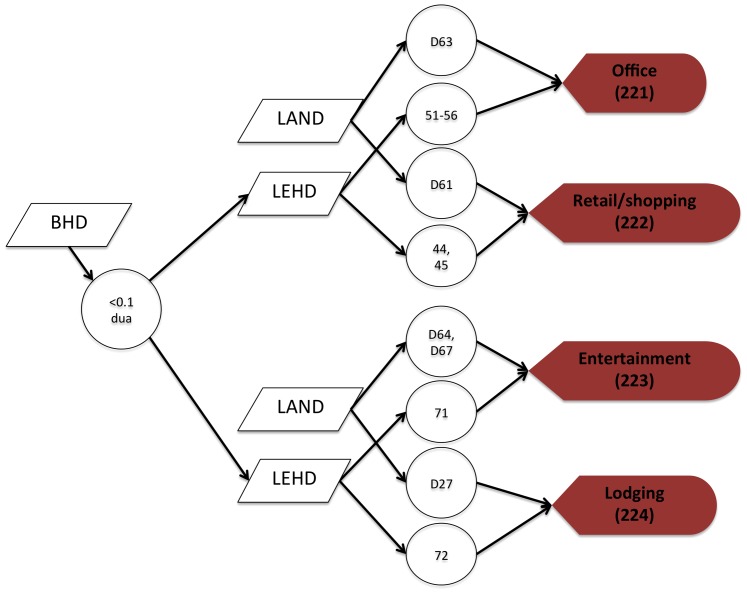
A flow chart depicting the process to map commercial land uses. Locations mapped as office commercial use were defined as an office building or office park by a census landmark polygon (LAND) or by North American Industry Classification System 2012 (NAICS) 2-digit code 51–56, which includes information, finance and insurance, real estate, professional, scientific and technical services, management of companies, and administrative and support services. Because commercial and residential uses are often mixed (e.g. particularly vertically, with commercial on the ground floor and residential above), and because the Longitudinal Employer-Household Dynamics (LEHD) data are mapped at the Census block-level, the residential housing density also had to be low (<0.1 dua). Consequently, areas that are mapped as urban residential land use could also contain some commercial land uses as well.

**Figure 3:**
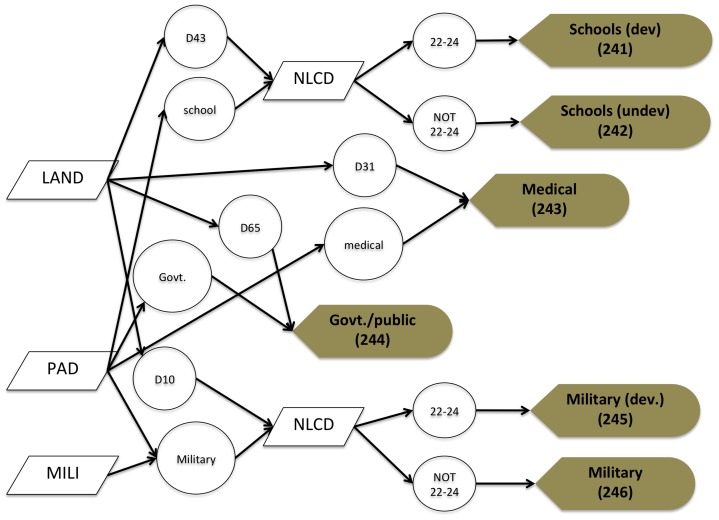
A flow chart depicting the process to map institutional land uses. Educational uses are mapped through Census landmark polygons (LAND) with values of D43 (Educational institution, including academy, school, college, and university) or if designated as a school in the Protected Areas Database US v1.3 (PAD). Educational institutions are further classified as developed (including buildings and playgrounds) using the National Land Cover Dataset 2006 (NLCD) with built-up values 22–24, or undeveloped (that includes grass fields and other vegetation types). Medical uses and government/public uses are mapped similarly, using LAND values  =  D31 (hospitals) or PAD designation as a hospital or other medical use; or D65 (government complex) or government designation from PAD. Military uses are depicted using LAND with values of D10 or as mapped in PAD or in the military polygons from the National Transportation Atlas Database 2010 (MILI). Developed portions of military lands, which can range from airports to storage facilities to residential uses, are differentiated using NLCD built-up values 22–24 (or if not, then are mapped as military undeveloped).

The sub-group and class levels within each of these four major groups further differentiate the primary land use or activity occurring in an area (Table 1), resulting in 69 different classes (plus an additional 11 water-based classes). It is important to note that interacting with the definition of classes (i.e. intensity) is the spatial scale or minimum mapping unit that land use is represented at – in this case the classification is attempting to be consistent with most 30 m resolution datasets (e.g., NLCD) – which is roughly 1 ha (nominally 9 cells). Using the four-group structure, I developed sub-group and classes building on Anderson level 2 codes as well as the typology defined by the North American Industrial Classification System (NAICS). Built-up uses are divided into residential, commercial, industrial, institutional, and transportation. Urban residential uses are defined at 1.6 dwelling units per acre (dua; 3.95 units per ha), based on the US Census definition of urban population of 1000 people per square mi [6], while the urban high category is greater than 10 dua based on typical densities at which public transportation is viable [23]. Suburban areas have residential densities below urban threshold but greater than 1 unit per 1 ha (0.4 dua), which is a typical density at which centralized services such as municipal sewer and water supply are provided versus de-centralized septic and water supply (e.g., ground water well) systems. Production classes include agricultural, mining, timber, energy, animal, and aquaculture and were designed to be roughly consistent with major categories of the Census of Agriculture, the NRI, and NAICS. Recreation classes distinguish uses that typify urban parks such as playgrounds, ball fields, golf courses, and courts, as opposed to less intense uses where natural land cover dominates. “Natural parks” are places that are managed primarily for recreation and occur in relatively natural land cover settings with many natural processes intact. These are typically accessible to the public, and can be further separated into motorized and non-motorized forms of recreation. A developed facility class is reserved for ski resorts, campgrounds, and the like. Facilities such as stadiums and amusement parks are included under the built-up group because they usually occur inside a building and are relatively passive activities. Conservation classes represent areas that are primarily for maintain “nature”. “Natural areas” are places where the primary use is to maintain natural vegetation and processes, such as nature reserves, research natural areas, and areas of critical environmental concern, as well as municipal watersheds. These areas usually are not accessible to the public. “Wildlife habitat” is an area that is primarily managed for hunting, fishing, and “watchable” opportunities, which often focus on big game and charismatic species. Areas designated as wilderness are placed in their own, distinct class. Finally, a miscellaneous class includes private land conservation easements, archaeological and historical sites, and other miscellaneous values. Maps that reflect conservation classes such as cemeteries are derived largely from land ownership and management datasets. Figures 1, 2, 3 illustrate the datasets and rules used to classify these classes.

**Table 1:** A multi-purpose, hierarchical land use classification system.

Level 1	Level II	Level III	Code
**1 Water**	**Natural - area**	**Lake**	**111**
		**Swamp/marsh**	**112**
		**Playa**	**113**
	**Human - area**	**Reservoir**	**121**
	**Natural - linear**	**River**	**131**
		**Wash**	**132**
	**Estuary**	**Estuary & complex channels**	**141**
	**Human - linear**	**Canal/ditch**	**151**
	**Wetlands**	**Wetlands**	**161**
	**Ocean**	**Open ocean**	**171**
		**Bay inlet**	**172**
**2 Built-up**	**Residential**	**Dense urban (>0.1 ac)**	**211**
		**Urban (0.1**–**1)**	**212**
		**Suburban (1**–**2.5 ac)**	**213**
		**Exurban (2.5**–**10 ac)**	**214**
		**Rural (10**–**40 ac)**	**215**
	**Commercial**	**Office**	**221**
		**Retail/shopping centers**	**222**
		**Entertainment (stadiums, amusement, etc.)**	**223**
		**Lodge**	**224**
	**Industrial**	**Factory, plant**	**231**
		**Landfill (land fill, waste trt.)**	**232**
		**Confined animal feeding**	**233**
		**Utilities (power, sewage)**	**234**
	**Institutional**	**Schools (dev)**	**241**
		**Schools (undeveloped)**	**242**
		**Medical (hospitals, nursing home, etc.)**	**243**
		**Government/public**	**244**
		**Military/DOD/DOE (dev)**	**245**
		**Military/DOD (training)**	**246**
		**Fire & police stations**	**247**
		**Church/religious**	**248**
		**Prison/penitentiary**	**249**
	**Transportation**	**Airports (developed)**	**251**
		**Highways, railways**	**252**
		**Other transportation**	**253**
		**Port, train station**	**254**
		**Undeveloped**	**255**
	**Miscellaneous**	**Rural buildings, cemetery**	**261**
**3 Production**	**General**	**General agricultural**	**310**
	**Cropland**	**Cropland/row crops**	**311**
		**Pastureland**	**312**
		**Orchards**	**313**
		**Sod & switch grass**	**314**
		**Aquaculture**	**315**
	**Rangeland**	**Grazed**	**321**
		**Stock tank**	**322**
	**Mining**	**Mining strip mines, quarries, gravel pits**	**330**
		**Mine shafts**	**331**
	**Timber**	**Timber harvest**	**341**
		**Timber plantations**	**342**
	**Extractive/barren land**	**Oil/Gas wells**	**351**
		**Misc. barren**	**352**
**4 Recreation**	**Undifferentiated park**	**General park**	**410**
	**Developed park**	**Urban park**	**411**
		**Golf course**	**412**
		**Motorized**	**413**
		**OHV staging area/trailhead**	**414**
		**Resort/ski area**	**415**
		**Marina**	**416**
		**Campground/ranger station**	**417**
		**Picnic/trailhead**	**418**
		**Boat/fishing access**	**419**
	**Natural park**	**Natural park**	**421**
		**Designated recreation area**	**422**
		**Designated scenic area**	**423**
**Conservation**	**Public**	**Wildlife habitat (hunting & fishing)**	**511**
		**Conservation area (BLM)**	**512**
		**Nature reserve**	**513**
		**Wilderness**	**514**
		**Areas of Critical Env. Concern, Research Natural Area**	**515**
		**Fish & Wildlife Service refuge**	**516**
		**Wilderness study area**	**517**
		**Archaeology, historic site, scenic area**	**518**
		**Wild & Scenic river**	**519**
	**Public-limited access**	**Municipal watershed**	**521**
		**Corps of Engineers dam**	**522**
		**Marine Protected Area**	**523**
	**Private easement**	**Wildlife conservation**	**531**
		**Agricultural conservation**	**532**

Note that because the variety of uses around land areas covered by water are quite complex and dynamic, here I simply distinguish naturally-occurring (e.g. lakes) from those that have are human-created (e.g. reservoir, canals). This simple distinction, however is quite useful and important, as previous efforts to examine the effects of human modification using land cover data alone have been forced to place all lakes and reservoirs wholly into either human modified or natural [24].

### Compile available datasets and construct map

I attempted to identify and compile a range of publicly-available spatial datasets that could be used to spatially represent the variety of land use classes defined in the classification system, that included a variety of aspects of housing characteristics, employment, recreational facilities, land cover, and transportation (Table 2). For the majority of these classes this was possible, but there remain a minority of classes that were not explicitly recorded in the mapped dataset because of data gaps. I then generated a series of simple rules to map each land use class, following the basic principles of von Thünen/Ricardo land rent theory [25], based on the economic notion of “highest and best use” that changes predictably with distance to markets and resources. In practice though, this dataset should be considered an initial attempt at estimating land use that should be critically evaluated, challenged, and improved on – as currently no other national extent, full sector, high-resolution land use dataset or classification exists. Note that currently the *primary use* is mapped at a given location, but in future versions the dataset could be extended to recognize that there can secondary or even tertiary uses at a given location (e.g., commercial uses mixed with residential).

Data are available at: http://csp-inc.org/public/NLUD2010_20140326.zip.

**Table 2:** Datasets used to construct the national land use database.

Group	Sub-Group	Datasets
Built-up	Residential	BHD = US Census 2010 block housing density (1:100k)
		LEHD = US Census Longitudinal Employer-Household Dynamics (block level, www.lehdmap.did.census.gov )
		NLCD = National Land Cover Dataset 2006 (30 m; www.mlrc.org; ) *
		PLC = US Census 2010 place polygons** (www.census.gov/geo/www/tiger)
	Commercial	
		
	Commercial	LAND = Census area landmarks (1:100k), LEHD
	Industrial	LAND, LEHD
	Institutional	MILI = Military lands from National Transportation Atlas 2010 (1:100k) (www.bts.gov/publications/national_transportation_atlas_database)
		PAD = Protected Areas Database-US (1:100k; http://gapanalysis.usgs.gov/padus/, v1.3)
		AIRP = US Census TIGER 2010 (1:100k)
	Transportation	BLKS = US Census 2010 blocks with 0 housing units, long and narrow
		TRDS = US Census TIGER 2010 roads, interstates and state highways
	Miscellaneous	PAD, general resource production activities
Production	General	NLCD, Cropland Data Layer (CDL; http://nassgeodata.gmu.edu/CropScape/)
	Cropland	NLCD
	Grazing	NLCD
	Timber	NLCD
	Mining, oil & gas	MINE = Active mines and plants in the US (USGS, 2005; www.mrdata.usgs.gov/mineplant)
Recreation	General	NLCD, PAD
	Urban park	LAND, PARK = US Census parks (1:100k)
	Natural park	LAND, PARK
	Developed faciility	INFRA = US Forest Service FS Topo Recreation Facilities (1:24k; www.fsgeodata.fs.fed.us/vector)
		SKI = Ski resort locations from National Weather Service (1:100k; www.nohrsc.noaa.gov/gisdatasets)
Conservation	Natural area	PAD, areas of critical environmental concern, research natural areas, wildlife refuges, etc.
	Wildlife habitat	PAD, wildlife habitat and state wildlife areas
	Wilderness	PAD, Wilderness areas
	Miscellaneous	PAD, other
Water	water	NHD = National Hydrographic Dataset (1:24k, areas, water bodies, nhd.usgs.gov)
		NID = National Inventory of Dams 2009 (1:24k, geo.usace.army.mil)

*With highways and roads filtered/removed

**Removed county, unknown and Census Designated places.

### Processing of datasets

All datasets were projected to Albers Equal Area Conic and converted to raster datasets (at 30 m). Data on the location of airports (AIRP) were obtained from the US Census Bureau (www.census.gov/geo/www/tiger) as simple polygonal boundaries. Block housing density (BHD) data were obtained from the US Census 2010. “Water blocks” (those identifying a lake/reservoir/river), as well as blocks with 0 housing units were removed. Data on census blocks occupied by transportation (BLKS) were obtained from the US Census 2010, and blocks with 0 housing units and those that intersected interstates and US highways were identified. These long, linear blocks demarcate transportation, typically interstate corridors and on/off-ramps.

Data on agricultural cropland were obtained from the Cropland Data Layer (CDL) from the National Agricultural Statistical Service (http://nassgeodata.gmu.edu/CropScape/). I grouped the CDL classes into three general agricultural classes. Cropland (row crops) was assigned to various crop types including grains and vegetables such as corn, cotton, rice, etc. Pasture land was assigned to alfalfa, pasture/grass, and hay types. An orchard class was assigned to tree and shrub-based production such as cherries, peaches, apples, oranges, etc. Finally, I assigned sod and switch grass to a miscellaneous agricultural land use class.

Data on various land mark features such as shopping malls, schools, etc. were obtained from the US Census 2010 (LAND). These features were re-coded in the following way based on the TIGER Census Feature Class Codes: D10 = military, D31 = hospitals, D37 = correctional facility, D43 = schools, D61 = commercial retail (malls, shopping centers), D63 =  office building or office park, D65 = government center; D67 = entertainment/stadium, D81 = golf courses, and D82 = cemetery. Additional data on military lands (MILI) was obtained from the National Transportation Atlas (2010; www.bts.gov/publications/national_transportation_atlas_database). Employment density and type data were obtained from the US Census Longitudinal Employer-Household Dynamics (LEHD; www.lehdmap.did.census.gov). These data provide the number of employees in each block, and using the 2-digit NAICS code I grouped these types into agricultural (11), commercial (22, 23, 44–45, 51–56, 71, 72, 81), industrial (21–23, 31–33, 42, 48–49), government (92), educational (61), health (62), and transportation (48–49). Recreational facilities on US Forest Service lands (INFRA) were mapped using data were obtained from the FS Topo Recreation Facilities (www.fsgeodata.fs.fed.us/vector). These points were buffered by 100 m.

Data on the location of active mines and processing plants (MINE) were obtained from the USGS Mineral Resources Data System (www.mrdata.usgs.gov/mineplant). Only records that were attributed as a current producer or plant were included. These locations were then buffered up to 400 m, but for only those locations mapped as Barren land (rock/sand/clay) in NLCD 2006. Water classes were derived from the US Geological Survey National Hydrography Dataset (high resolution, 1∶24,000; nhd.usgs.gov). I converted the areal-based features from NHD (i.e. lakes/reservoirs, wide-rivers, washes, and marsh/wetlands) into 30 m raster cells (center-method). Note that the water class from the NLCD 2006 was removed, so water classes in NLUD are only derived from the NHD data – providing a much more detailed, consistent, and precise representation of water features. This includes removing the water pixels from the original NLCD that are offshore from the coastline feature class from NHD. To distinguish natural water features (e.g., lakes and rivers) from human-made (e.g., reservoirs), I identified the NHD water body features that contained “reservoir” in the GNIS name or if it was the nearest feature (up to 200 m) from a dam identified in the National Inventory of Dams 2009 (NID; www.geo.usace.army.mil).

Land cover data were obtained from the 2006 NLCD (www.mrlc.org). These data were used to differentiate developed from undeveloped areas on institutional land using NLCD values of 21–24 on military or institutional (educational) land use types, then these locations were changed to schools (developed) or military (developed) NLUD classes. Wetland areas are identified partially from NLCD as well as from the NHD. Note that the NLCD data were filtered to remove a number of artifacts, including thin lines of developed areas (primarily along rural roads caused by “burning in” pixels from a highway data layer) that were removed if they were less than 90 m wide and were within 90 m of an interstate, highway, or county highway (from US Census 2010 TIGER roads). If a pixel was removed, it was filled in with the class of the nearest “natural” cover type using Anderson code level I (e.g., grass, shrub, forest, wetland).

Data on land ownership and management were obtained from the US Geological Survey's Protected Areas Database-US (PAD) v1.3 (http://gapanalysis.usgs.gov/padus). I assigned land use classes based on unique values (n = 1119) from the field “Primary Location Description” and by interpreting information from additional fields such as management type, GAP status code, and name of protected area (a look up table is available on request). I also used morphological analysis to filter small and thin “sliver” polygons, and well as filled in “gaps” between polygons that were caused primarily because of the state-by-state nature of the PAD-US dataset. Data on mostly urban parks (PARK) were obtained from US Census landmark data as well as from the PAD dataset. Data on urban or city areas were obtained from the US Census 2010, using the “places” geography. These polygons were used to exclude certain land uses, such as campgrounds and exurban residential development. Locations of ski resorts (SKI) was obtained from the National Weather Service (www.nohrsc.noaa.gov/gisdatasets) and were buffered by 200 m.

### Evaluation

It is difficult to rigorously evaluate the results of this dataset because of the differences of it with other datasets in terms of class definitions, the scale of data (county to state vs. very fine), time differences (2007 vs. 2010), and coverage or geographic extent (e.g., NRI is only for privately-held lands). I do, however, provide a general comparison of the results to the NLCD as well as to the NRI. In addition, I validated the NLUD against a detailed land use dataset that was generated by interpreting land uses from recent (2005–2010, mostly 2007–8) high-resolution (<1 m) aerial photography sampled at ∼6,000 random locations across mainland US. For each sample location or “chip” (roughly 600 m×600 m), a trained photo interpreter mapped polygons of each land use type following an established protocol [26]. I intersected the NLUD data with the chips, such that each of the 1.9 million cells (30 m) that intersected a chip polygon was assigned the underlying land use to assess against the NLUD value.

## Results

**Figure 4:**
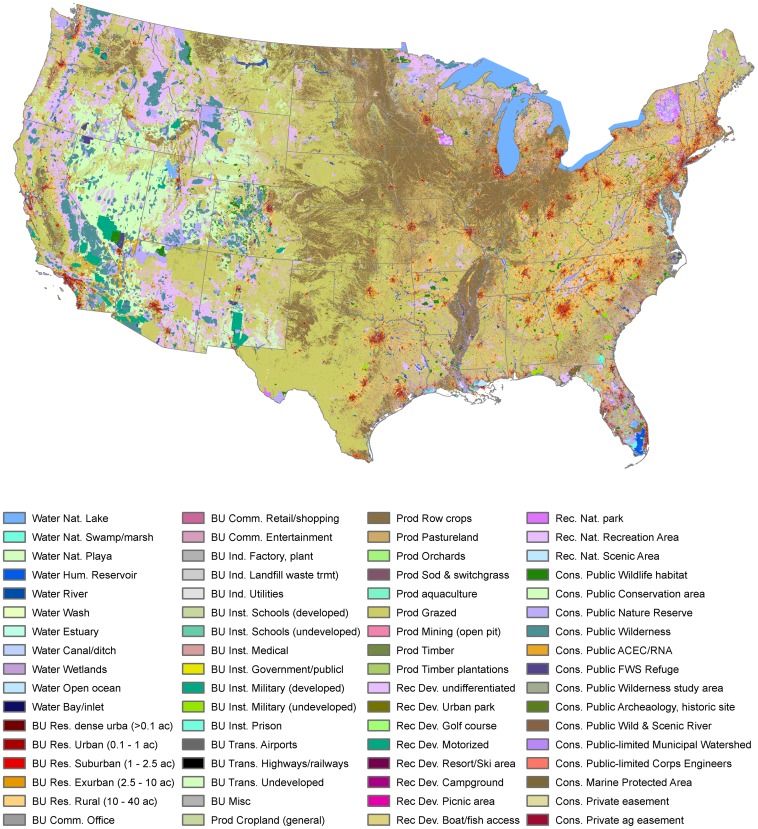
The National Land Use dataset for 2010, depicting 59 land use classes for the conterminous US, at a resolution of 30(0.09 hectares).

The final land use classification system consisted of 5 groups, 26 sub-groups, and 79 classes (Table 3; Figure 4). I found that data were insufficient to map 11 classes (e.g., energy, animal hunt & trapping, fish ponds, algal beds, confined animal feeding operations, etc.), so that the first version of the dataset contained 58 classes. Built-up areas occupy 13.6% of mainland US, but that the majority of this occurs as low-density exurban/rural residential (9.1% of the US), while more intensive built-up land uses occupy 4.5%. For every acre of urban and suburban residential land, there are 0.13 commercial, 0.07 industrial, 0.48 institutional, and 0.29 acres of interstates/highways. Areas of resource production occupy the majority of the US at 52%, with 20.0% in cropland. Recreational activities occupy 10%, including 0.14% in urban parks and 0.08% in golf courses. Conservation lands occupy 16%, with 3% of the mainland US in designated wilderness areas. Water (although not a land use *per se*) occupies 6% with natural lakes and rivers (including large portions of the Great Lakes) occupying 1.2%, human constructed facilities of reservoirs and canals occupy 0.78%, wetlands/swamps occupy 3.5%, and playas/washes occupy 0.3%.

**Table 3:** Area and proportion of the US for sub-group level of the National Land Use dataset 2010.

Group	Sub-group	Area (km^2^)	Proportion of US
Built-up	Residential – Urban	148,502	1.83%
	Residential – rural	732,797	9.05%
	Commercial	20,540	0.25%
	Industrial	11,044	0.14%
	Institutional	71,529	0.88%
	Institutional undeveloped	24,403	0.30%
	Transportation	43,698	0.54%
	Misc. open space	49,592	0.61%
Production	Cropland	1,637,198	20.21%
	Miscellaneous	16,160	0.20%
	Grazing	2,559,409	31.60%
	Mining	938	0.01%
	Timber	20,436	0.25%
Recreation	General	756,049	9.33%
	Urban park	11,202	0.14%
	Golf course	6,813	0.08%
	Natural park	39,683	0.49%
Conservation	Wildlife habitat	190,358	2.35%
	Reserves & conservation	818,840	10.11%
	Wilderness	243,077	10.11%
	Watershed	19,897	3.00%
	Private easements	33,325	0.25%
Water	Natural	250,027	0.41%
	Human	61,094	3.09%
	Wetlands, swamps, playas	301,444	0.75%
	Oceanic	31,813	3.72%

**Figure 5:**
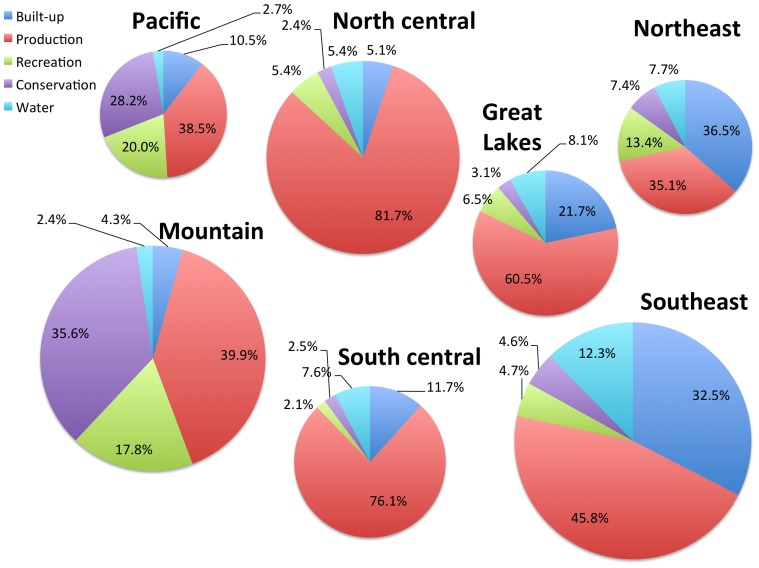
Regional variation of the proportion of major land uses in the conterminous US. The relative extent of 7 regions is depicted by the size of the pie-charts: Pacific (CA, OR, WA); Mountain (AZ, CO, ID, MT, NM, NV, UT, WY); North central (IA, KS, MN, MO, ND, NE, SD); South central (AR, LA, OK, TX), Great Lakes (IL, IN, MI, OH, WI), Northeast (PA and northeastern states), Southeast (DE, KY, WV and southern states). Land in productive uses dominated the major land uses for all regions but the Northeast, which was narrowly dominated by built-up uses. The proportion of land in recreation and conservation land uses exceeded 10% for the Pacific, Mountain, and Northeast regions. Note: “water” is not a land use per se, and includes both water and wetland cover types.

There are important geographic variations in the land use patterns (Figure 5). Not surprisingly, built-up areas are more extensive in the eastern US (36.5% northeast and 32.5% southeast) and minimal in the west (4.3% mountain and 5.1% in North-central). Production areas are most extensive in the North-central region (81.7% especially croplands) and the South central region (76.1% especially grazing) and minimal in the Northeast (35.1%). Recreational uses are most extensive in the Pacific (20.0%) and minimal in the South-central region (2.1%). Conservation lands are most extensive in the Pacific and Mountain regions (28.2% and 35.6%), and minimal in the North-central and South-central (2.4% and 2.5%).

The results of the accuracy assessment of NLUD against the validation land use dataset were promising (Table 4), although I was unable to validate the recreation and conservation groups because the validation dataset did not adequately distinguish these land use types. The overall accuracy and the group (top) level was 84.2% for built-up and 99.3% for agricultural. At the sub-group level, Residential was correctly classified 74.4% (primarily confused with commercial). Commercial was correctly classified 25.6%, being largely confused with Institutional sub-group. All other sub-groups (Industrial, Transportation, and Miscellaneous) were >91% accurate.

**Table 4:** Results of the accuracy assessment testing the National Land Use dataset against a sample-based, detailed land use dataset.

Group	Sub-group	Water		Built-up						Agricultural		
		Natural	Human	Residential	Commercial	Industrial	Institutional	Transportation	Misc	Cropland	Grazing	Timber
**Water**	**Natural**	18,551	-									
	**Human**		13,078			2						
**Built-up**	**Residential**		69	55,958	272		2			59	2	
	**Commercial**			9,644	1,100							
	**Industrial**		6	655	416	528		44				37
	**Institutional**				2,465		510					
	**Transportation**			5,384				475				
	**Misc**								458			
**Agri-cultural**	**Cropland**			27	11	2				441,237		32,736
	**Grazing**										442	
	**Mining**		391	1,121			1					
**Recreation**			18	2,410	28		7					
**Unknown**			596	14	4					70		3,108

		Water		Built-up						Agricultural		
Water		Natural	Human	Residential	Commercial	Industrial	Institutional	Transportation	Misc	Cropland	Grazing	Timber
	**Natural**	100.00%										
**Built-up**	**Human**		96.40%			0.40%						
	**Residential**		0.50%	74.40%	6.30%		0.40%				0.50%	
	**Commercial**			12.80%	25.60%							
	**Industrial**			0.90%	9.70%	99.20%		8.50%				0.10%
	**Institutional**				57.40%		98.10%					
	**Transportation**			7.20%				91.50%				
**Agri-cultural**	**Misc**								100.00%			
	**Cropland**			0.00%	0.30%	0.40%				100.00%		99.90%
	**Grazing**										99.50%	
	**Mining**		2.90%	1.50%	0.00%		0.20%					
**Recreation**			0.10%	3.20%	0.70%		1.30%					
**Unknown**			4.40%	0.00%	0.10%					0.00%		9.50%

**Figure 6:**
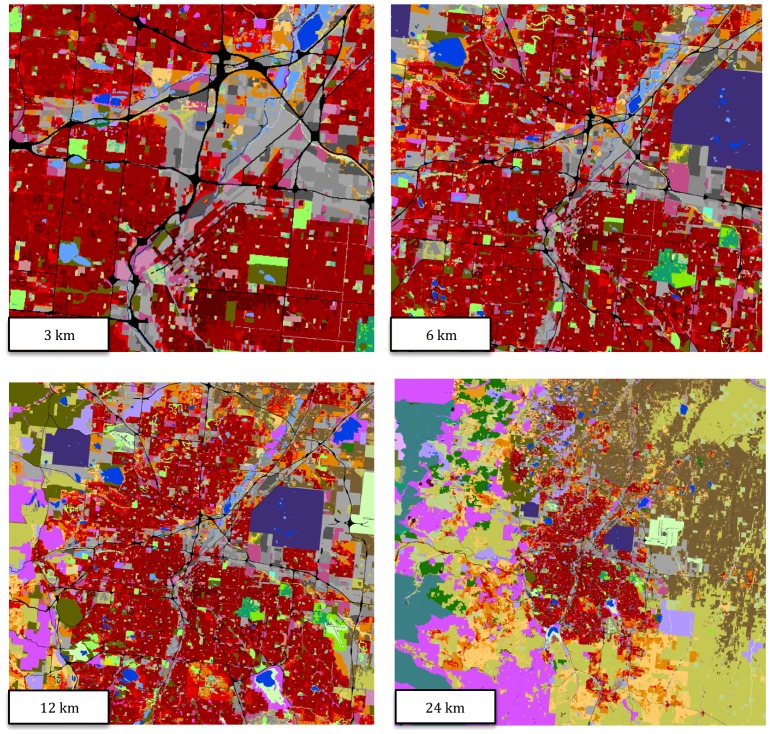
The National Land Use dataset for 2010 centered on Denver, Colorado (I-25 and I-70), showing NLUD at 4 different scales. Detailed industrial (light grey), commercial (dark grey), and transportation (black) land uses can be readily scene in the detailed map (3 km scale), while regional land use patterns surrounding the Denver metro area, including “open space” lands composed of recreational, conservation, and production lands, are shown in the coarse map (24 km scale). The full legend is provided in Figure 4.

This new map of land use provides a comprehensive, detailed, and spatially-explicit characterization of the primary human activities that are carried out on US lands (Figure 6). In addition to a partial validation, it is useful to compare the NLUD to two existing land use datasets. The first is a comparison of NLUD built-up uses to the NLCD 2006 (Table 5). Although NLCD is one of the three primary input datasets used to create NLUD, there are some important differences. I found that residential urban was predominately identified in the urban cover classes (81.0%), but that 12.9% of urban residential cells were (mis-) identified as forest cover. For exurban and rural residential land uses less than 1 unit per 2.5 acres (0.025–0.4 dua), NLCD identified only 8.9% in the urban cover classes, with over 57.2% identified as forested land cover, and 33.8% in agricultural, grass, and shrubland cover classes. This concurs with previous findings that NLCD misses significant areas of the US that are in low-density residential land use [6], [7].

**Table 5:** A comparison of the National Land Use database (2010) against the National Land Cover Dataset (2006) for built-up uses.

	Water	Urban				Barren	Forest	Shrub	Grass	Ag.	Wetland
NLUD Built-up	1	21	22	23	24	3	4	5	7	8	9
Res. Urban	0.00%	27.40%	37.30%	13.90%	2.40%	0.00%	12.90%	2.10%	2.40%	1.50%	0.00%
Res. Rural	0.00%	6.80%	1.80%	0.30%	0.10%	0.00%	57.20%	8.90%	9.30%	15.60%	0.00%
Commercial	1.30%	10.90%	11.10%	24.40%	19.50%	1.40%	9.20%	5.20%	3.80%	13.00%	0.20%
Industrial	1.30%	11.40%	10.80%	21.40%	16.90%	1.10%	10.00%	4.80%	4.00%	18.20%	0.10%
Institutional	0.90%	3.60%	16.10%	20.20%	9.10%	0.30%	22.50%	4.40%	2.10%	5.00%	15.80%
Inst. Undevel.	0.10%	3.60%	0.30%	0.10%	0.10%	6.60%	12.40%	62.30%	10.20%	2.00%	2.30%
Transportation	0.20%	35.80%	29.20%	12.70%	4.70%	0.20%	3.90%	2.80%	2.60%	7.10%	0.90%
Miscellaneous	1.30%	8.70%	5.90%	10.10%	4.60%	1.50%	18.80%	15.60%	8.00%	24.70%	0.80%

A second comparison is to the NRI dataset. The NRI found that in 2007 about 5.74% of the US was classified as developed land uses (that includes built-up lands permanently removed from rural land use, including urban and rural transportation corridors), which compares roughly to an estimated 6.26% from the NLUD using the built-up group and urban park & golf course classes (minus the low-density exurban class of less than 0.1 dua. Roughly adjusting the NRI estimate to be concordant in time with the 2010 NLUD, the NRI estimate would increase to about 5.87% (the US population increased from 2007 to 2010 by 1.0236%, assuming a linear relationship between population increase and developed land area). In the land use dataset, the built-up group is not limited to private land, and a very small amount of built-up (mostly classed as institutional or industrial) occurs on public lands, which might account for some of the gap. Comparing intensive agricultural land use, the NRI estimated 24.54% in cropland and pastureland uses, as compared to 20.9% in the land use dataset. A smaller area might likely occur because the land use dataset agricultural categories are derived primarily from satellite imagery, and consequently the narrow strips of adjacent lands (fallow, corners of center pivot sections, etc.) that are likely in agricultural use might be classified as grassland, shrubland, or perhaps barren in the NLCD. The definition of the other main NRI classes (rangeland and forestland) are simply too different from the land use dataset to provide a reasonable comparison, primarily because activities that occur on these types, such as grazing, can occur on both rangelands and forested land, as well as on both public and private land.

### Example applications

**Figure 7:**
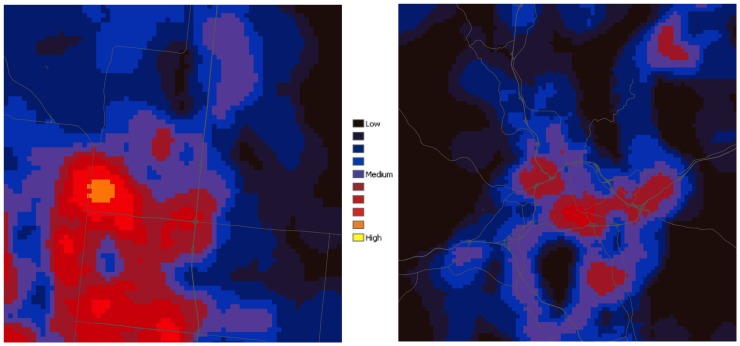
The diversity of land uses shown for Fort Collins, CO (left) and Ashville, NC (right). Higher diversity is depicted in yellow/red, with lower diversity shown in blue and dark blue. Major highways and interstates are labeled for reference.

In addition to investigating patterns and summaries at local, regional or state-levels, these data can be used to examine many different types of questions, particularly those that aim to quantify the condition of metropolitan and urban areas (Figure 7). For example, the smart growth literature has identified a key measure of lower energy use and higher “walkability”, called the land use mix, which is commonly calculated at project-level extents using parcel-level data [22]. I measured this using an entropy index of the diversity of land uses within 1 mile:
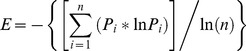
where *P_i_* is the proportion of use type *i* and *n* = 4. Land use classes were combined to reflect types *i*  =  live (residential), work (commercial, industrial, & institutional), play (recreation), and shop (retail commercial). This index shows interesting patterns of land use mix (Figure 7) delineating more mixed-use areas from more homogenous parts of a city, or comparing urban areas (e.g., Fort Collins, Colorado and Ashville, North Caroline had a mean land use mix value of 0.498 and 0.229, respectively. Locations with higher land use diversity have been found to be strongly related to “walkability” of a neighborhood as well as lower vehicle miles travelled [23].

**Figure 8:**
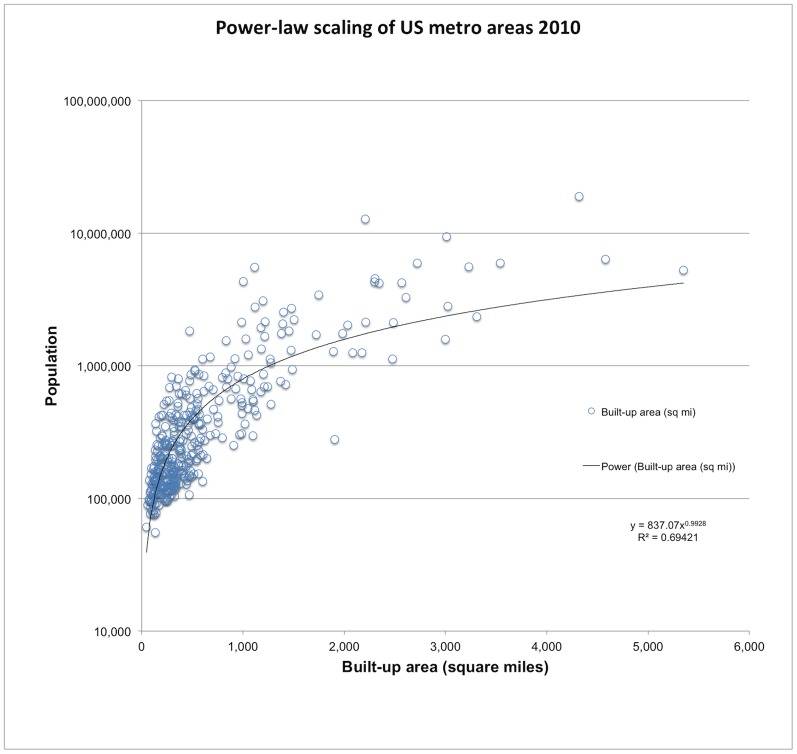
The population of US metropolitan areas follows a power law-scaling to the developed “footprint”, which includes built-up land uses except for urban parks and golf courses. Computing the ratio of the raw population to the footprint (scale) adjusted population provides a relative measure of land use efficiency for metropolitan areas.

A second application is to more precisely estimate the power-law scaling of urban areas [27]. Discussions about urban sprawl have been confounded by poor aggregate indicators such as population density, which do not account for overall population size. I found that the population of US metropolitan areas scaled to the developed footprint: y = 972.75 X^0.974^, R^2^ = 0.68, where *X* is defined as any built-up land use except urban parks/golf courses (Figure 8). Refining earlier work [28], this equation can be used to allow comparison amongst metro areas to allow fairer and more consistent examination of potential efficiencies of resource use (or conversely, “sprawl”). By taking the ratio of the current population to the scale-adjusted population, a relative indication of how efficiently (per capita) land is being used, compared to all other metro areas. For example, of metro areas with 2010 populations of Ashville, NC had an index of 0.46, suggesting that in 2010 its pattern was less efficient than average, whereas Fort Collins, CO was a bit better than average (1.27) and Santa Barbara, CA was well above average (2.76).

A third example is identifying potential conflict areas with natural resources such as the agricultural-urban interface, which has become increasingly contentious and has engendered numerous right to farm acts across the US. Using NLUD I found that in 2010 roughly 13% of intense agricultural lands (cropland and pastureland, 222,610 km^2^) were within ½ mile of the developed footprint (of at least 160 acres in area, a reasonable proxy for minimum farm size). Similarly, about 10.5% of the forested ecosystems were within the wildland-urban interface (within ∼½ mile of developed lands). I also found that 13% of the (mainland) US coastline was developed.

## Discussion

Although the dataset constructed here nominally represents conditions in 2010, the datasets compiled actually represent a range of time frames. The primary datasets of housing density, employment, and transportation do represent 2010 conditions, but land cover is from 2006. The other datasets represent 2010 conditions (mostly used in the buil-up group — landmarks, institutional, airports, military, etc.), while the recreation and conservation groups represent conditions that range from early 2000s to 2010. This requires a bit of interpretation, particularly for the conservation classes. Another opportunity is to update the land use dataset when the 2011 version of NLCD 2011 becomes available (anticipated in early 2014), or potentially generate a series of national land use datasets to be consistent with the NLCD series (2001, 2006, 2011).

Both the level of detail in the land use classes, and the high-resolution of the dataset make it particularly vulnerable to potential misclassifications and errors. The general accuracy of the NLUD at sub-group levels is on par with other previous efforts (e.g., NLCD 2006; [29]), but represents a substantial improvement over urban/built-up classes of NLCD (84% vs. 56%) and is roughly similar to agricultural cover types (77 and 88%).

The greatest uncertainty in mapping land use centers on the uses in the grazing/rangeland sector of agriculture, and recreation, and conservation on public lands. This is in part because these are multi-use lands, where multiple uses are occurring simultaneously (e.g., many national forest lands provide grazing, recreation, and timber, and national parks have a dual mission to preserve resources and to provide for the enjoyment of parks). It is also due to the relatively crude surrogate used — ownership/management from the Protected Areas Database — which could be improved by incorporating more detailed information from resource and travel management plans. Similarly, because many of the parks and protected areas are represented solely as their ownership boundaries, the variety of uses within a park typically are not differentiated.

Notably, the grazing land use class has a high degree of uncertainty, as it was mapped simply as privately owned lands dominated by shrub and grass land cover. Many privately-owned, conserved lands could also be incorporated into the NLUD, such as from the recently-created National Conservation Easement Database.

Key findings from this study are that built-up areas occupy 13.6% of mainland US, but that the majority of this occurs as low-density exurban/rural residential (9.1% of the US), while higher-density built-up land uses occupy 4.5%. For every acre of urban and suburban residential land, 0.13 commercial, 0.07 industrial, 0.48 institutional, and 0.29 acres of interstates/highways. Three example applications of the dataset were provided, but there are a wide variety of additional applications and uses of this dataset.
